# Bagging survival tree procedure for variable selection and prediction in the presence of nonsusceptible patients

**DOI:** 10.1186/s12859-016-1090-x

**Published:** 2016-06-07

**Authors:** Cyprien Mbogning, Philippe Broët

**Affiliations:** Université Paris-Saclay, Univ. Paris-Sud, UVSQ, CESP, INSERM, 14-16 Avenue Paul-Vaillant Couturier, Villejuif, 94807 France; Abirisk consortium WP4, 14-16 Avenue Paul-Vaillant Couturier, Villejuif, 94807 France; Faculty of Medicine, Univ. Paris-Sud, 63 Rue Gabriel Péri, Le Kremlin-Bicêtre, 94276 France; Assistance Publique - Hôpitaux de Paris, Hôpital Paul Brousse, 14-16 Avenue Paul-Vaillant Couturier,, Villejuif, 94807 France

**Keywords:** Bagging, Survival tree, High-dimensional data, Nonsusceptible individuals, Genomic

## Abstract

**Background:**

For clinical genomic studies with high-dimensional datasets, tree-based ensemble methods offer a powerful solution for variable selection and prediction taking into account the complex interrelationships between explanatory variables. One of the key component of the tree-building process is the splitting criterion. For survival data, the classical splitting criterion is the Logrank statistic. However, the presence of a fraction of nonsusceptible patients in the studied population advocates for considering a criterion tailored to this peculiar situation.

**Results:**

We propose a bagging survival tree procedure for variable selection and prediction where the survival tree-building process relies on a splitting criterion that explicitly focuses on time-to-event survival distribution among susceptible patients.

A simulation study shows that our method achieves good performance for the variable selection and prediction. Different criteria for evaluating the importance of the explanatory variables and the prediction performance are reported. Our procedure is illustrated on a genomic dataset with gene expression measurements from early breast cancer patients.

**Conclusions:**

In the presence of nonsusceptible patients among the studied population, our procedure represents an efficient way to select event-related explanatory covariates with potential higher-order interaction and identify homogeneous groups of susceptible patients.

## Background

Since the inception of large-scale genomic technologies, there has been a growing interest in analyzing the prognostic and predictive impact of high-dimensional genomic markers. However, the extremely large number of potential interaction terms prevent from being specified in advance and incorporated in classical survival models. In this context, tree-based recursive partitioning methods such as CART (Classification And Regression Tree [1]) provide well-suited and powerful alternatives. This nonparametric methodology partitions recursively the predictor space into disjoint sub-regions (so-called terminal nodes or leaves) that are near homogeneous according to the outcome of interest. This framework is particularly well-suited to detect relevant interactions and produce prediction in high-dimensional settings.

Since the first extension of CART to censored data (termed as survival trees) proposed by Gordon and Olshen [2], many new methods have been proposed so far (for a review see [3]). Broadly speaking, the key components for a survival tree are: the splitting criterion, the prediction measure, the pruning and tree selection rules. The splitting survival-tree criteria rely either on minimizing the within-node homogeneity or maximizing the between-node heterogeneity. They are based on various quantities such as the distance between Kaplan-Meier survival curves [2], likelihood-related functions (e.g. [4]) or score statistics (e.g. [5]) such as weighted or unweighted Logrank test statistics. The final prediction measure, within each terminal node, is typically based on non-parametric estimations of either the cumulative hazard function or the survival function. The pruning and selection rules are applied to find the appropriate subtree and avoid overfitting.

However, the well-known instability of tree-based structures has led to the development of so-called survival ensemble methods such as bagging survival tree and random survival forest [6, 7]. The main idea is that the combination of several survival tree predictors has better predicting power that each individual tree predictor. The general strategy is to draw bootstrap samples from the original observations and to grow the maximal tree for each of these samples. This strategy also circumvents the problem of pruning and selection since each tree is grown full size. The final prediction is obtained by averaging the predictions from each individual trees. In practice, the bagging can be viewed as a special case of random survival forests where all the covariates are considered as relevant candidates at each node. These methods also provide a way to define various variable importance measures that can be used for variable selection.

Even though survival trees are non-parametric methods, their constructions rely heavily on the chosen model-related splitting criteria that are based on either parametric or semi-parametric modeling assumptions (e.g. [4, 8]). Thus, for a particular problem, the choice of the splitting criteria is crucial to the performance of the tree regarding variable selection and prediction [9]. This problem is particularly appealing in the context of survival data with nonsusceptible individuals where the investigator is interested in identifying homogeneous subgroups according to the time-to-event outcome among the individuals who are susceptible to experience the event of interest. In clinical oncology, these nonsusceptible individuals (sometimes referred as long-term survivors or cured patients) are those who have been successfully cured from the disease by the primary treatment. For infectious and immune diseases, these individuals are those who are resistant to certain pathogens or tolerant to specific antigens. In such mixed population, none of the classically used splitting criteria explicitly focuses on the time-to-event survival distribution among susceptible individuals, which raises some open questions about their performance.

In the literature, various survival models taking into account for a fraction of nonsusceptible patients (also called “improper survival distribution” models) have been proposed. The oldest framework relies on two-component mixture models which explicitly assumes that the population under study is a mixture of two subpopulations of patients (susceptible/nonsusceptible) in a parametric or semi-parametric modeling approach (for a review, see [10]). A different framework proposed more recently defines the cumulative hazard risk as a bounded increasing positive function that can be interpreted from either a mechanistic model (as first introduced by [11] in oncology) or a latent activation scheme [12].

In this work, our aim is to unravel complex interactions between genomic factors that act on the time-to-event distribution among susceptible patients while adjusting for the confounding effect associated to the existence of a fraction of susceptible patients in the population under study.

Thus, we propose a bagging survival tree procedure for variable selection and prediction which is tailored to this situation. The strategy relies on an improper survival modeling which considers a linear part for taking into account for known confounders associated with the nonsusceptible fraction and a tree structure for the event-related explanatory variables. The building of the survival trees rely on a model-based splitting criteria that explicitly focuses on susceptible patients. The considered splitting criterion is linked to a recently proposed model-based discrimination index that quantifies the ability of a variable to separate susceptible patients according to their time-to-event outcome [13].

Next, the splitting criteria and the general procedure are presented. We then compare the results obtained with this procedure to those obtained with the classical Logrank statistic as the splitting criteria. We illustrate the clinical interest of this procedure for selection and prediction among patients with early-stage breast carcinoma for whom gene expression measurements have been collected. We conclude with a discussion on the practical use of the procedure, its limitations and the potential extensions.

## Methods

### Notations and improper survival model

Let the continuous random variables *T* and *C* be the true event and censoring times. Let *X*=*min*(*T,C*) be the observed time of follow-up, *δ*=1_(*X*=*T*)_ the indicator of event and *Y*(*t*)=**1**_(*X*≥*t*)_ the at risk indicator at time *t*. Here, we consider that for nonsusceptible individuals *T*=*∞*_+_. Thus, the survival function *S*(*t*) of *T* is said to be improper with *S*(*∞*_+_)>0. The hazard function (or the instantaneous event rate) of *T* is noted: *λ*(*t*)=*f*(*t*)/*S*(*t*), where *f*(*t*) is the density function of *T*. The corresponding cumulative hazard function is noted $\Lambda (t)={\int _{0}^{t}}\lambda (s)ds$ with a finite positive limit *θ* such as *Λ*(*t*=*∞*_+_)=*θ*<*∞*_+_. Let *Z*=(*Z*_1_,*Z*_2_) be the (*m*_1_+*m*_2_)-dimensional vector of covariates where *Z*_1_ is the *m*_1_-dimensional sub-vector of known confounding covariates linked to the nonsusceptible state and *Z*_2_ is the *m*_2_-dimensional sub-vector of explanatory covariates of interest (associated with the time-to-event outcome).

For each patient *i* (*i*=1,…,*n*), the observed data consists of (*X*_*i*_,*δ*_*i*_,*Z*_*i*_). We assume noninformative censoring for *T* and *C* [14]. For modeling the time-to-event survival distribution, we propose to consider the following tree-structured improper survival model:

$$S(t|Z_{i})=\exp \left[ -\Lambda \left(t|Z_{1i},W_{il}^{\Phi(Z_{\mathbf{2}})}\right) \right] $$

where the bounded cumulative hazard function $\Lambda \left (t|Z_{1i},W_{il}^{\Phi (Z_{\mathbf {2}})}\right)$ depends on *Z*_1_ and *Z*_2_ through a linear and a tree component, respectively. In this latter case, the dummy covariate $W_{il}^{\Phi (Z_{\mathbf {2}})}$ is such as $W_{il}^{\Phi (Z_{\mathbf {2}})}=1$ if the *i*^*th*^ observation belongs to the *l*^*th*^ leave (or terminal node) of the tree *Φ*(*Z*_**2**_) and zero otherwise.

Here, the cumulative hazard function is modeled such as: $\Lambda \left (t|Z_{1i},W_{il}^{\Phi (Z_{\mathbf {2}})}\right)=\theta e^{\alpha ^{T}Z_{1i}}\left \{1-\exp \left [ -H \left (t;W_{il}^{\Phi (Z_{\mathbf {2} })}\right) \right ] \right \} $ where *H*(*t*) is an unspecified continuous positive function increasing from zero to infinity which formulates the shape of the time-to-event survival distribution for each terminal node. Thus, the cumulative hazard function $\Lambda \left (t|Z_{1i},W_{il}^{\Phi (Z_{\mathbf {2}})}\right)$ is bounded, increases with *t* and reaches its maximum for $\phantom {\dot {i}\!}\theta e^{\alpha ^{T}Z_{1i}}$ where *α* is an unknown vector of parameters associated to *Z*_1_ and *θ* is a positive parameter.

At any split, if we assume proportionality between the two child nodes with *Z*^∗^ a binary variable for node membership, the previous model can be written in terms of the hazard function such as:

1 $$ \lambda (t|Z_{i})=\theta e^{\alpha Z_{1i}}h(t)e^{\gamma Z_{i}^{\ast }}e^{-H(t)e^{\gamma Z_{i}^{\ast }}}   $$

where $h(t)=\frac {\partial H\left (t\right) }{\partial t}$ and *γ* is an unknown parameter associated with variable *Z*^∗^.

### Splitting criterion

The classical use of Logrank related statistics in survival trees relies on the fact that these statistics are considered as between-node heterogeneity criteria.

In the context of a mixed population (nonsusceptible/susceptible), we have proposed [13] a pseudo-R2 criterion that can be interpreted in terms of percentage of separability obtained by a variable according to time-to-event outcomes of susceptible patients. This criterion represents a good candidate for the splitting process.

In the following, we give the formula of the splitting criterion through its relationship with the partial log-likelihood score.

Let (*X*_*i*_,*δ*_*i*_,*Z*_*i*_; *i*=1,…,*m*; *m*≤*n*) be the set of observed data within node *τ*. We consider splitting the parent node *τ* of size *m* into two child nodes *τ*_*L*_ and *τ*_*R*_. Let ${ Z_{i}^{\ast }}$ be a binary variable such as ${Z_{i}^{\ast }}=1$ if the *i*^*th*^ observation belongs to node *τ*_*L*_ and zero otherwise, and *γ* the unknown parameter associated with *Z*^∗^. The partial likelihood based on (1) is as follows:

$$L(\gamma,\alpha)=\prod_{i=1}^{m}\left[ \frac{e^{\alpha Z_{1i}}e^{\gamma Z_{i}^{\ast }}e^{-H(X_{i})e^{\gamma Z_{i}^{\ast }}}}{ \sum_{j=1}^{m}Y_{j}(X_{i})e^{\alpha Z_{1j}}e^{\gamma Z_{j}^{\ast }}e^{-H(X_{i})e^{\gamma Z_{j}^{\ast }}}}\right]^{\delta_{i}}. $$

The score vector deduced from the partial log-likelihood for the improper survival model (1) under the hypothesis of *γ*=0 is such as:

$${} \begin{aligned} U &= \left\{ \frac{\partial \log L}{\partial \gamma }\mid_{_{\gamma =0}}\right\}\\ &=\sum_{i=1}^{m}\delta_{i}w(X_{i})\left({Z_{i}^{\ast }}-\frac{ \sum_{l=1}^{m}Y_{l}(X_{i})e^{\alpha Z_{1l}}{Z_{l}^{\ast }}}{ \sum_{l=1}^{m}Y_{l}(X_{i})e^{\alpha Z_{1l}}}\right) \end{aligned} $$

with *ω*(*X*_*i*_)=1−*H*(*X*_*i*_). Here, *H*(*t*)=−*log*(1−*Λ*_0_(*t*)/*θ*), where *Λ*_0_(*t*) is a baseline cumulative hazard function bounded by *θ* under the hypothesis of *γ*=0. It is worth noting that when *θ* tends to infinity (the nonsusceptible fraction tends to zero) then *ω*(*X*_*i*_) tends to one. In this latter case, the proposed score corresponds to the classical adjusted Logrank statistic which is appropriate for proper survival model.

The corresponding robust variance estimator [15] is such as:

$${} V=\sum_{i=1}^{m}\left\{ \begin{array}{c} \delta_{i} \omega \left(X_{i}\right) \left({Z_{i}^{\ast }}-\frac{\sum_{l=1}^{m}Y_{l}(X_{i})e^{\alpha Z_{1l}}{Z_{l}^{\ast }}}{\sum_{l=1}^{m}Y_{l}(X_{i})e^{\alpha Z_{1l}}}\right) \\ -\sum_{l=1}^{m}\frac{\delta_{l}\omega \left(X_{l}\right) }{\sum_{r=1}^{m}Y_{r}(X_{l})e^{\alpha Z_{1r}}}\left({Z_{i}^{\ast }}-\frac{\sum_{r=1}^{m}Y_{r}(X_{l})e^{\alpha Z_{1r}}{Z_{r}^{\ast }}}{\sum_{r=1}^{m}Y_{r}(X_{l})e^{\alpha Z_{1r}}}\right) \end{array} \right\}^{2}. $$

The practical expression of *U* and *V* are obtained by replacing *Λ*_0_, *θ*, and *α* by their respective estimators $\hat {\Lambda }_{0}$, $\hat {\theta }$, and $\hat {\alpha }$. Here, $\widehat {\Lambda }_{0}$ is the left-continuous version of the Breslow’s estimator [16, 17]. The estimated quantity $\widehat {\theta }$ is equal to $\hat {\Lambda }_{0}(t_{\max })$ where *t*_max_ is the last observed failure time and $ \widehat {\alpha }$ is the maximum partial likelihood estimator of *α* under the null hypothesis (*γ*=0).

The quantity $S=\frac {U^{2}}{V}/K$ where *K* is the total number of distinct event times is a pseudo-R2 measure [13]. This criterion is unit-less, ranges from zero to one and increases with the effect of the splitting variable. It is also not affected by the censoring, the sample size and the nonsusceptible fraction. To a factor *K*, this criterion can also be interpreted as the robust score statistic obtained from the partial log-likelihood under the improper survival model [15].

### Bagging procedure and prediction estimate

We consider a learning set $\mathcal {L}$, consisting of *n* independent observations: $\mathcal {L}=\{{(X_{i},\delta _{i},Z_{i})},\ i=1,\ldots,n\}$. Let $\mathcal {L}_{b}^{\ast }$ (*b*=1,…,*B*) denotes the b^th^ bootstrap sample of the training set $\mathcal {L}$ obtained by drawing with replacement *n* elements of $\mathcal {L}$. According to random sampling of observations with replacement, an average of 36.8 *%* are not part of $\mathcal {L}_{b}^{\ast }$. Let *OOB*$_{b}= \mathcal {L}\setminus \mathcal {L}_{b}^{\ast }$ be the set of these elements. The observations in *OOB*_*b*_ are not used to construct the predictor $\mathcal {P}_{b}$; they constitute for this predictor the so-called Out Of Bag (*OOB*) sample.

The bagging procedure is as follows:

1. Repeat for *b*=1,…,*B*
  Take a bootstrap replicate $\mathcal {L}_{b}^{\ast }$ of the training set $\mathcal {L}$
  Build a survival tree such as: ∗ For each split candidate variable *Z*^∗^ (based on the information from *Z*_2_) compute the corresponding splitting criterion *S*(*Z*^∗^) presented above. ∗ Do the same procedure for all the split candidate variables. ∗ Find the best split *S*^∗^ which is the one having the maximum value over all the candidates. Then, a new node is built and the observations are splitted accordingly. ∗ Iterate the process until each node reaches a pre-defined minimum node size or be homogeneous. ∗ Construct the final tree denoted $\mathcal {T}^{b }\left (W^{b }\right) $ where $W_{l}^{\left (b\right)} (l = 1,\ldots,L(b))$ is a vector of indicator variables representing the *L*(*b*) leaves of the tree such that $W_{il}^{\left (b\right)} = 1$ if the *i*^*th*^ observation belongs to the *l*^*th*^ terminal node of $\mathcal {T}^{b }$, and 0, otherwise.
  Calculate the cumulative hazard function (CHF) estimator for each terminal leave of each bootstrap tree $\mathcal {T}^{b}$. ∗ The Breslow-type estimator of the baseline cumulative hazard [16, 17] in a terminal node *l* of the tree $\mathcal {T}^{b}$ is computed as
    $${} \begin{aligned} \hat{\Lambda}_{l}^{b}\left(t\right) &=\hat{\Lambda}\left(t|W_{l}^{\left(b\right) }=1\right)\\ &=\sum_{i;t_{i}\leq t}^{n}1_{\left\{ W_{il}^{\left(b\right) }=1\right\} }\left(\frac{\delta_{i}}{\sum_{j = 1}^{n}1_{\left\{ W_{jl}^{\left(b\right) }=1\right\} }{Y_{j}^{l}}\left(t_{i}\right) e^{\hat{\alpha}Z_{1j}}}\right) \end{aligned} $$
    where $\hat {\alpha }$ is the partial log-likelihood estimator obtained using all the learning data from the tree $\mathcal {T}^{b }$. ∗ The Nelson-Aalen estimator of the baseline cumulative hazard [18, 19] in a terminal node *l* of the tree $\mathcal {T}^{b }$ is computed as:
    $${} \begin{aligned} \hat{\Lambda}_{l}^{b}\left(t\right) &=\hat{\Lambda}\left(t|W_{l}^{\left(b\right) }=1\right)\\ &=\sum_{i;t_{i}\leq t}^{n}1_{\left\{ W_{il}^{\left(b\right) }=1\right\} }\left(\frac{\delta_{i}}{\sum_{j = 1}^{n}1_{\left\{ W_{jl}^{\left(b\right) }=1\right\} }{Y_{j}^{l}}\left(t_{i}\right) }\right) \end{aligned} $$
2. Compute the CHF prediction estimator:
  The CHF prediction estimator for a new patient *j* with covariate *Z*_*j*_ is computed as follows. The patient’s covariates $Z_{2_{j}}$ are dropped down each tree. Then, the prediction is obtained as the weighted average of the estimated CHF over the learning datasets with the same membership terminal node assignment than the new case:
  $$\hat{\Lambda}\left(t\right| Z_{j}) = \frac{1}{B}\sum_{b = 1}^{B} \sum_{l = 1}^{L(b)}1_{\left\{ W_{jl}^{\left(b\right)} = 1\right\} }e^{\hat{\alpha}Z_{1_{j}}}\hat{\Lambda}_{l}^{b}\left(t\right) $$
  where *L*(*b*) is the number of leaves nodes of the tree $\mathcal {T}^{b}$

### Measures of prediction accuracy

Various measures have been proposed so far for assessing the estimated survival predictions (e.g. [20, 21]). One of the most popular in censored data analysis is the integrated Brier score [22] which is now widely used in survival tree-based methods. The Brier score is interpreted as the mean square error between the estimated survival function and the data weighted by the inverse probability of censoring. Its square root can be interpreted as the expected distance between the predicted risk and the true event status. The Brier score is a pointwise measure which is given at time *t* by:

$$\begin{aligned} BS(t) = \frac{1}{N}\sum_{i=1}^{N}&\left[\hat{S}\left(t|Z_{i}\right)^{2}\hat{G}^{-1}(X_{i})1_{\left(X_{i}\leq t,\delta_{i}=1\right)}\right.\\ &\quad\left. +\left[ 1-\hat{S}\left(t|Z_{i}\right) \right]^{2}\hat{G}^{-1}(t)1_{\left(X_{i} > t\right)} \right] \end{aligned} $$

where $\hat {G}(t)$ is the nonparametric Kaplan-Meier estimate of the censoring distribution which represents the weights in the expected Brier score.

The integrated Brier score over time is given by:

$$IBS = \frac{1}{\max (X_{i})}\int_{0}^{\max (X_{i})}BS\left(t\right) dt. $$

Here, we take advantage of the bagging strategy that provides OOB CHF estimator (2) for computing the Out Of Bag IBS denoted by *IBS*^∗^. This latter quantity is obtained such as:

$$IBS^{\ast} = \frac{1}{\max (X_{i})}\int_{0}^{\max (X_{i})}BS^{\ast}\left(t\right) dt,  $$

where

$$\begin{aligned} BS^{\ast}\left(t\right) &= \frac{1}{N}\sum_{i=1}^{N}\left[\hat{S}^{\ast}\left(t|Z_{i}\right)^{2}\hat{G}^{-1}(X_{i})1_{\left(X_{i}\leq t,\delta_{i}=1\right)} \right.\\ &\left.\quad+\left[ 1-\hat{S}^{\ast}\left(t|Z_{i}\right) \right]^{2}\hat{G}^{-1}(t)1_{\left(X_{i} > t\right) } \right] \end{aligned} $$

with $\hat {S}^{\ast }\left (t|Z_{i}\right) = \exp \left (- \hat {\Lambda }^{\ast }\left (t|Z_{i}\right) \right)$ being the OOB predicted survival function for individual *i* at a given time *t*. This internal validation procedure avoids the time-consuming cross-validation. Lower values of *IBS*^∗^ indicate better predictive performances.

For computing the *IBS*^∗^, the *OOB* prediction of the CHF is computed such as: Let 1_*i,b*_ equal one if the patient *i* is an *OOB* observation for the *b*^*th*^ bootstrap tree $ \mathcal {T}^{b}$, and zero otherwise. The *OOB* cumulative hazard function estimator for *i* is obtained by averaging only over bootstrap tree samples in which individual *i* is excluded.

2 $$ \hat{\Lambda}^{\ast }\left(t|Z_{i}\right) = \frac{\sum_{b=1}^{B}1_{i,b}\sum_{l = 1}^{L(b)}1_{\left\{ W_{il}^{\left(b\right) }=1\right\} }e^{\hat{\alpha}Z_{1_{i}}}\hat{\Lambda}_{l}^{b}\left(t\right)}{\sum_{b=1}^{B}1_{i,b}}   $$

### Importance score

The choice of a measure of importance for a variable can rely on either the prediction capacity or the discriminative ability of the variable through the tree structure. Here, we consider the following importance scores.

#### Index importance score (IIS)

For each bootstrap tree $\mathcal {T}^{b}$ indexed by *b*=1,…,*B*, let *ν*^*b*^ be a given node for the tree $\mathcal {T}^{b}$. For each component *j* of the vector *Z*_2_ and for each tree $\mathcal {T}^{b}$, the Importance Score of $Z_{2_{j}}$ is computed as the sum of the values of the splitting criterion at each split relying on this variable ($\phantom {\dot {i}\!}S_{\nu ^{b}}$) times the number of events in the split $(\Delta _{\nu ^{b}})\phantom {\dot {i}\!}$. This latter quantity corresponds to the value of the robust Logrank score under the improper survival model.

$${\omega_{j}^{b}}=\sum_{\nu^{b}\in \mathcal{T}^{b},\ \nu^{b} \text{is based on} Z_{2_{j}}} \Delta_{\nu^{b}} \times S_{\nu^{b}}. $$

These scores are summed across the set of trees, and normalized to take values between 0 and 100, with sum of all scores equal to 100:

$$IIS_{j}={\frac{1}{\kappa }}\sum_{b=1}^{B}{\omega_{j}^{b}} $$

where $\kappa ={\frac {1}{100}}\sum _{b,~j}{\omega _{j}^{b}}$.

#### Depth and index importance score (DIIS)

The second criteria is inspired from the Depth Importance measure that has been introduced by Chen et al. [23]. This measure is similar to the Index Importance Score but also considers the location of the splitting.

If *d*_*t*_ denotes the depth of the split of node *ν*^*b*^ in the tree $ \mathcal {T}^{b}$, we define

$$\omega_{j}^{\ast b}=\sum_{\nu^{b}\in \mathcal{T}^{b},\ \nu^{b} \text{is based on} Z_{2_{j}}} 2^{-d_{t}} \times \Delta_{\nu^{b}} \times S_{\nu^{b}}. $$

These scores are summed across the set of trees and normalized to sum to 100:

$$DIIS_{j}={\frac{1}{\kappa^{\prime }}}\sum_{b=1}^{B}\omega_{j}^{\ast b} $$

where $\kappa ^{\prime }={\frac {1}{100}}\sum _{b, ~j}\omega _{j}^{\ast b}$.

#### Permutation prediction importance score (PPIS)

The permutation importance is conceptually the most popular measure of importance for ensemble methods which relies on prediction accuracy. It is assessed by comparing the prediction accuracy of a tree before and after random permutation of the predictor variable of interest. For each tree $\mathcal {T}^{b}, b = 1,\ldots,B $ of the forest, consider the associated Out Of Bag sample *OOB*_*b*_. Let denote $IBS_{b}^{\ast }$ the OOB Integrated Brier Score based on the sample *OOB*_*b*_ and using the single tree $\mathcal {T}^{b}$ as predictor. The $IBS_{b}^{\ast }$ corresponds to a restriction of *IBS*^∗^ on the sample *OOB*_*b*_ (of cardinality equal to |OOB_*b*_|) using the predictor $\mathcal {T}^{b}$:

$$IBS_{b}^{\ast} = \frac{1}{\max (X_{i}, i \in OOB_{b})}\int_{0}^{\max (X_{i},~ i \in OOB_{b})}BS_{b}^{\ast}\left(t\right) dt $$

with

$${} \begin{aligned} BS_{b}^{\ast}\left(t\right) = \frac{1}{\left\vert \text{OOB}_{b}\right\vert }\sum_{i \in \text{OOB}_{b}}&\!\left[\!\hat{S}_{b}^{\ast}\left(t|Z_{i}\right)^{2}\hat{G}^{-1}(X_{i})1_{\left(X_{i}\leq t,\delta_{i}=1\right)}\right.\\ &\left.+\left[\!1-\hat{S}_{b}^{\ast}\left(t|Z_{i}\right)\! \right]^{2}\!\hat{G}^{-1}(t)1_{\left(X_{i} > t\right)}\! \right]. \end{aligned} $$

Then, for each component *j*=1,…,*m*_2_ of the vector $Z_{2} =(Z_{2_{1}},\dots,Z_{2_{m2}}) $ of predictors, the values $z_{2_{ij}}$ are randomly permuted within the *OOB*_*b*_ samples, and the prediction accuracy $IBS_{b}^{\ast j}$ is computed once again. The Permutation Importance is the average of increase in prediction error over the *B* bootstrap samples:

$$PPIS(Z_{2_{j}})={\frac{1}{B}}\sum_{b=1}^{B}\left(IBS_{b}^{\ast j} - IBS_{b}^{\ast}\right). $$

Large values of PPIS indicate a strong predictive ability whereas values close to zero indicate a poor predictive ability. In the following, we will denote *PPIS-NA* and *PPIS-BRE* the scores obtained using the Nelson-Aalen and the Breslow estimators, respectively.

### Basket of important variables

For selecting a subset (hereinafter referred as a basket) of the most important variables, the main problem is to choose a threshold value for the previous scores. Several performance-based approaches have been proposed in the literature to deal with the variable selection in Random Forests comparing either OOB or cross-validated errors of a set of nested models. Most of these procedures share the same methodological scheme and differ only in minor aspects (for a few see [24–26]). However, for survival data there is no consensus about which measure of prediction error is the most appropriate. Thus, each measure leads to a particular estimation of the prediction error that ultimately leads to select different subset of variables. Rather than using performance-based approaches, we propose hereafter to consider a strategy based on a testing procedure using a topological index which allows to select a basket of important variables.

In the following and without loss of generality, we suppose that the index score of interest is the IIS.

We then consider a permutation test at a global level *α* for testing the hypothesis

$$ \mathcal{H}_{0j} \ :\ IIS_{j} = 0 \ \textit{v.s} \ \ \ \mathcal{H}_{1j} \ :\ IIS_{j} \neq 0; \ j = 1,\ \ldots,\ m_{2}.  $$

The procedure consists in iterating between the following steps:

- Step 1: Use the learning set $\mathcal {L}$ to build the bagging predictor as describe in “Bagging procedure and prediction estimate” Section. Compute for each competing variable $Z_{2_{j}}$ the index score of importance *IIS*_*j*_ as describe in “Importance score” Section.
- Step 2: Let *σ*:{1,…,*n*}→{1,…,*n*} be a random permutation of the set {1,…,*n*}; let $\mathcal {L}_{\sigma } = \{{(X_{\sigma (i)},\delta _{\sigma (i)},Z_{1\sigma (i)}, Z_{2i})},\ i=1,\ldots,n\}$ be a partial permutation of $\mathcal {L}$. Use the learning set $\mathcal {L}_{\sigma }$ to build another bagging predictor using the same procedure as in the first step and compute again for each competing variable $Z_{2_{j}}$ the index score of importance $ II{S_{j}^{0}} $.
- Step 3: Repeat Step 2 a number *Q* of times.
- Step 4: Compute the P-values for each competing variable $Z_{2_{j}}$ as follows:
  $$p_{j} = \frac{1}{Q}\sum_{q = 1}^{Q} \mathbf{1}_{\{IIS_{jq}^{0} \geq IIS_{j}\} } $$
- Step 5: Using a Bonferroni procedure for multiple comparisons, the selected variables are those fulfilling the conditions *p*_*j*_≤*α*/*m*_2_; *j*=1,…,*m*_2_.

This procedure is conceptually similar to the one proposed by [27] to correct the bias of the so-called *Gini* importance in a Random-Forest framework. Nevertheless, in our framework, we have to take into account the covariables *Z*_1_ associated to the nonsusceptible individuals. For this purpose, the permutation scheme used in *Step 2* ensures that the existing relationship between the time-to-event observations and the covariates *Z*_1_ is not distorted under the null hypothesis.

## Results and discussion

### Simulation scheme

In order to evaluate the performance of the bagging survival strategy relying either on the classical adjusted Logrank splitting criterion (denoted LR) or the proposed pseudo-R2 criterion (denoted R2), we performed a simulation study as follows.

The data were generated from an improper survival tree using the following model:

3 $$\begin{array}{@{}rcl@{}}{} S(t|Z_{1}\ ;\ Z_{2}) & = & S\left(t|G_{1}\ ;\ G_{2},\ldots,G_{5}\right)  \\ & = & \exp [- \Lambda(t|G_{1}\ ;\ G_{2},\ldots,G_{5})]  \\ & = & \exp \left\{ -\theta e^{\alpha G_{1}}\left[ 1 - \exp\left(-\lambda_{0} t e^{~g(\gamma,G_{2},\ldots,G_{5})} \right) \right] \right\},  \end{array} $$

where

$$\begin{array}{@{}rcl@{}}{} \begin{array}{ll} g(\gamma,G_{2},\ldots,G_{5}) &= \gamma_{1}\mathbf{1}_{G_{2}=0,G_{3}=0}+\gamma_{2}\mathbf{1}_{G_{2}=0,G_{3}=1}+\gamma_{3}\mathbf{1}_{G_{2}}\\ & =1, G_{4}\,=\,{0} +\gamma_{4}\mathbf{1}_{G_{2}=1,G_{4}=1,G_{5}=0}+\gamma_{5}\mathbf{1}_{G_{2}}\\ & =1,G_{4}=1,G_{5}={1} \end{array} \end{array} $$

with *e*^*α*^=1.25, *λ*_0_=1, *γ*_1_=0.6, *γ*_2_=1.8, *γ*_3_=0.45, *γ*_4_=0.35, *γ*_5_=2. The Bernoulli variables *G*_1_,…,*G*_5_ related to the time-to-event variable *T* are generated using the following scheme:

$G_{i}\sim \mathcal {B}(\nu _{i}), \text {\ for\ }i = 1,\dots,5$ with *ν*_1_=0.5; *ν*_2_=0.6; *ν*_3_=0.5; *ν*_4_=0.3; *ν*_5_=0.65.

**Fig. 1:**
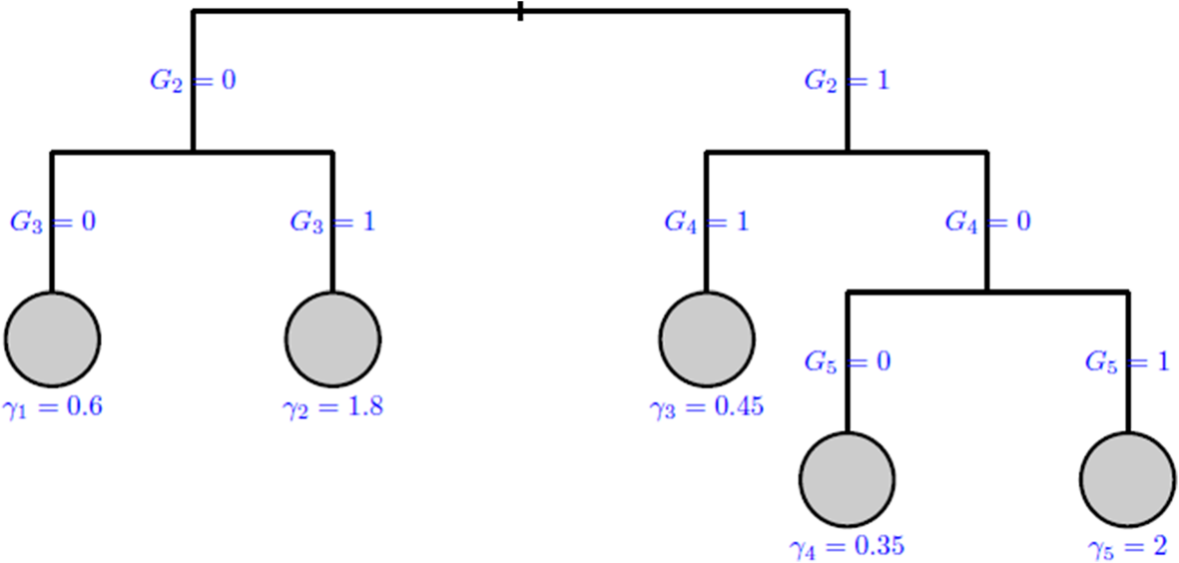
Simulated improper survival tree: The leaves are represented by circles and the number beneath each leaf node represents the log-hazard ratio of the underlying risk set

Predictor *G*_1_ is associated with the nonsusceptible fraction while predictors *G*_2_,…,*G*_5_ are associated to the survival distribution of the susceptible fraction through a five risk group survival tree. The underlying improper survival tree is displayed in Fig. 1. The censoring distribution was exponential with parameter chosen to give 10 and 25 *%* of censoring within the susceptible population. The parameter *θ* is such as exp(−*θ*) corresponds to the proportion of nonsusceptible individuals for the reference group (*G*_1_=0).

We considered eight different scenarios with, for each, three different values for the number of noise or non-informative covariables (10, 100 and 500), that are independent Bernoulli variables with *π*=0.5. Thus, a total of 24 different simulation sets were generated. The first four scenarios are based on model (3), with *N*=250 individuals, a proportion of nonsusceptible patients of 25 and 50 *%*, and the rate of censoring observations within the susceptible population of 10 and 25 *%*. The last four scenarios are also based on model (3), but with *N*=500 individuals and the same setting as the previous ones.

The simulation scheme is summarized in Table 1 where “censoring” represents the proportion of censoring among susceptible individuals and “plateau” the proportion of nonsusceptible individuals in the population.

**Table 1:** Simulation scenarios for the evaluation of the importance scores and the prediction accuracy

Scenario	*N*	plateau	censoring	Noise covariables
1 (a)				10
1 (b)	250	25 *%*	10 *%*	100
1 (c)				500
2 (a)				10
2 (b)	250	25 *%*	25 *%*	100
2 (c)				500
3 (a)				10
3 (b)	250	50 *%*	10 *%*	100
3 (c)				500
4 (a)				10
4 (b)	250	50 *%*	25 *%*	100
4 (c)				500
5 (a)				10
5 (b)	500	25 *%*	10 *%*	100
5 (c)				500
6 (a)				10
6 (b)	500	25 *%*	25 *%*	100
6 (c)				500
7 (a)				10
7 (b)	500	50 *%*	10 *%*	100
7 (c)				500
8 (a)				10
8 (b)	500	50 *%*	25 *%*	100
8 (c)				500

For all scenarios, LR and R2 are adjusted criteria for the known confounding factor G1 linked to the nonsusceptible state. We also evaluate the prediction accuracy using either the Nelson-Aalen (denoted NA) or the Breslow (denoted BRE) estimators. For prediction accuracy, we present the results obtained from the integrated Brier score (IBS). For the variable selection, we present the results obtained with IIS, DIIS and PPIS criteria.

For each scenario, we have generated 50 data sets. The bagging procedure with 400 trees was then applied to each data set with the two proposed splitting criteria. We then obtained 50 estimates of the Out Of Bag Integrated Brier Score for each method and each scenario.

We considered an additional scenario designed to mimic a data set that would reflect a situation, such as the one presented in our example, where variables are functionally related through groups (e.g. biological pathway). In practice, we generated correlated variables divided in five blocks of various sizes (ranging from 10 to 30 %) with correlations ranging between −0.2 to 0.3. We considered a situation with 500 individuals, a proportion of nonsusceptible patients of 25 *%*, a rate of censoring observations within the susceptible population of 10 and 25 *%* and two different values for the number of non-informative covariables (100 and 500).

#### Simulation results

**Fig. 2:**
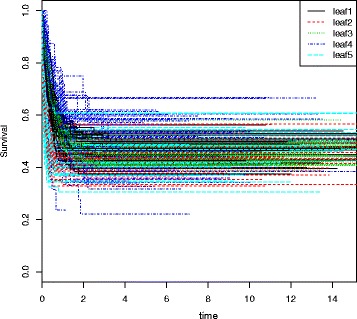
Kaplan-Meier curves of the 50 generated datasets for the different leaves

Figure 2 shows for one scenario and the 50 generated datasets, the Kaplan-Meier curves obtained for the different leaves.

#### Prediction results

**Fig. 3:**
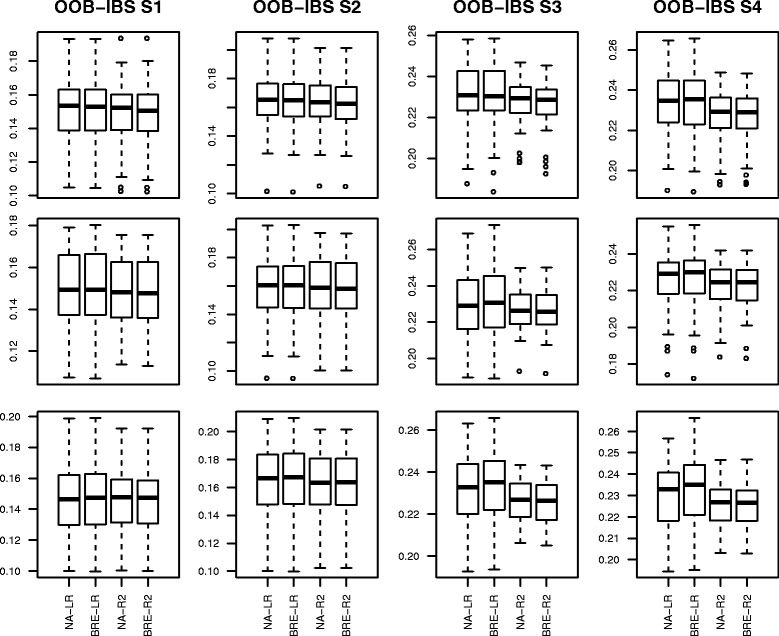
Box-plot of the Out Of Bag Integrated Brier Score on simulated data set for scenarios 1–4: first column represents scenarios 1a–1c; second column represents scenarios 2a-2c; third column represents scenarios 3a–3c and fourth column represents scenarios 4a–4c

**Fig. 4:**
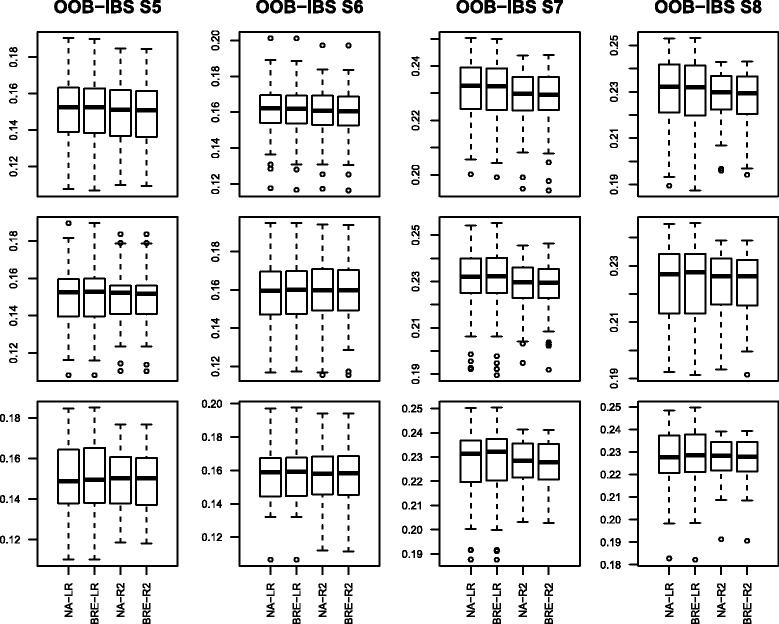
Box-plot of the Out Of Bag Integrated Brier Score on simulated data set for scenarios 5–8: first column represents scenarios 5a–5c; second column represents scenarios 6a–6c; third column represents scenarios 7a–7c and fourth column represents scenarios 8a–8c

The Box-plots of the 50 values of OOB-IBS are presented in Figs. 3–4 corresponding to scenarios 1–4 and 5–8 respectively.

In the first scenario (first column from left to right of Fig. 3), the OOB-IBS are consistently and slightly lower than their counterparts of scenario 2 (second column from left to right of Fig. 3). This was expected because of the increase in censoring proportion among the susceptible population from 10 *%* in scenario 1 to 25 *%* in scenario 2.

The OOB-IBS obtained using our proposed “pseudo-R2” splitting criterion are better (lower median value with a smaller variability) than those obtained with the stratified Logrank criterion. The “Pseudo-R2” consistently outperforms the Logrank in term of prediction accuracy for the first two scenarios. For these scenarios, the results obtained with BRE and NA estimators are comparable. The impact of the additional noise variables on the prediction accuracy seems insignificant.

The same remarks can be made for scenarios 3 and 4 using the last two columns from left to right of Fig. 3. The only additional information here is an increase of the global magnitude of the OOB-IBS from the first two columns of Fig. 3 to the last two columns. This is mainly due to the decrease of the proportion of susceptible population from 75 *%* in scenarios 1–2 to 50 *%* in scenarios 3-4, leading to a decrease of the number of events observed. These scenarios are more challenging than the previous ones.

The results of scenarios 5–8 (Fig. 4) are slightly better than those of scenarios 1–4. This is mainly due to the increase in the number of individuals from 250 to 500.

**Fig. 5:**
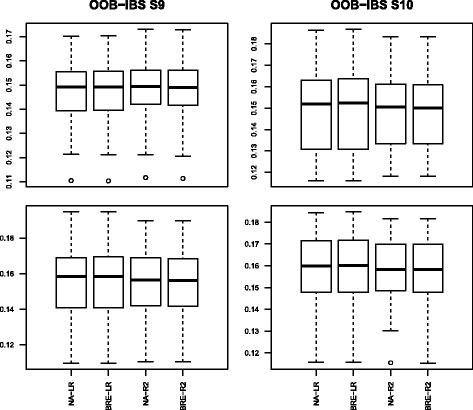
Box-plot of the Out Of Bag Integrated Brier Score on simulated data set for the additional scenario with correlated variables divided in five correlated blocks of various sizes

In the additional scenario with correlated variables (Fig. 5), the results are comparable to those of scenarios 5 and 6. The “Pseudo-R2” criterion still has an edge on the Logrank criterion in term of prediction accuracy.

#### Importance scores results

**Fig. 6:**
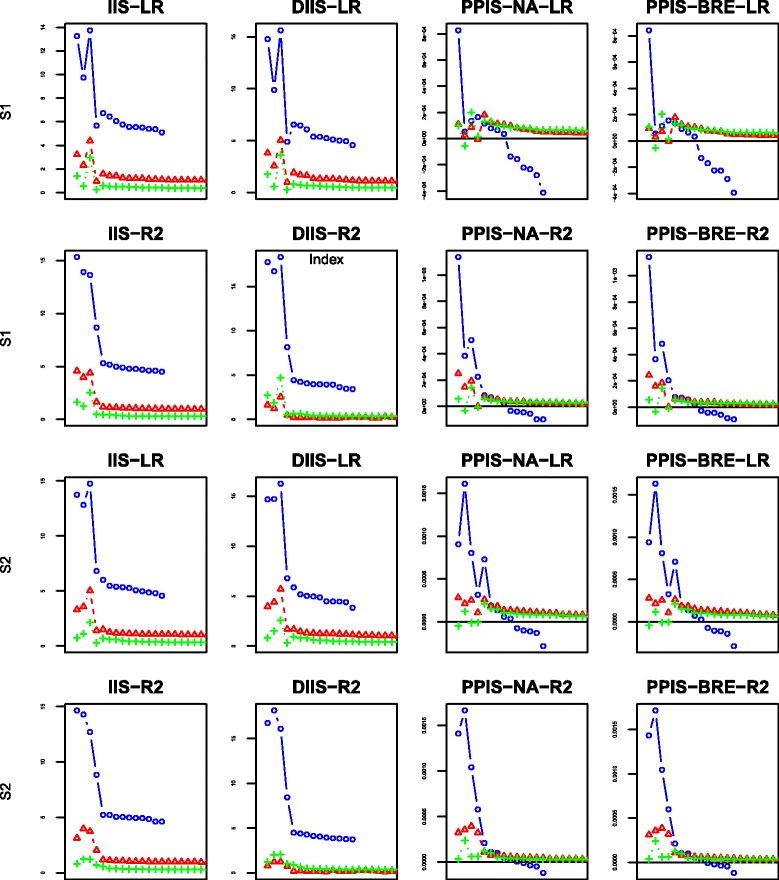
Variable importance results for scenarios 1–2: the first two rows from the top to the bottom represent scenario 1 while the last two represent scenario 2; ∘ represents 10 noise variables, *Δ* 100 noise variables and + 500 noise variables; “LR” represents the adjusted Logrank splitting criterion and “R2” the Pseudo-R2 splitting criterion

**Fig. 7:**
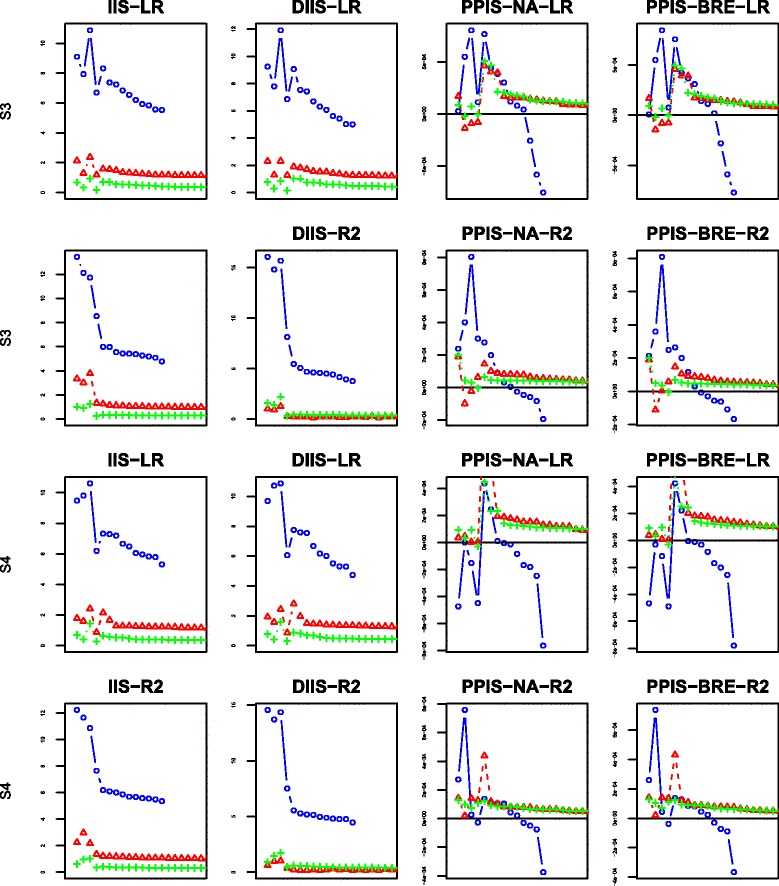
Variable importance results for scenarios 3–4: the first two rows from the top to the bottom represent scenario 3 while the last two represent scenario 4; ∘ represents 10 noise variables, *Δ* 100 noise variables and + 500 noise variables; “LR” represents the adjusted Logrank splitting criterion and “R2” the Pseudo-R2 splitting criterion

**Fig. 8:**
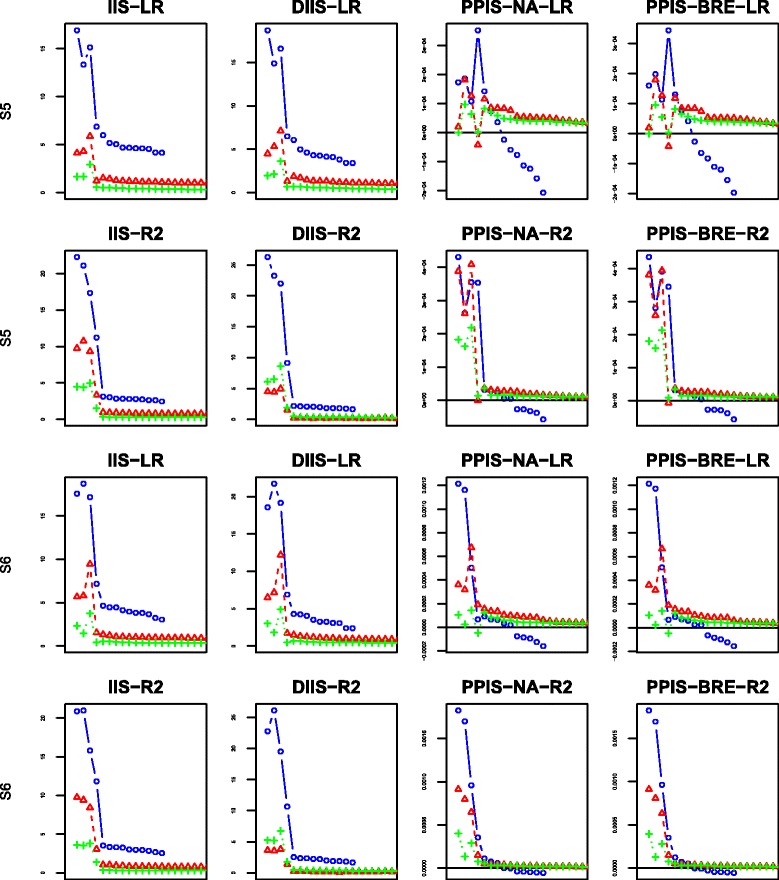
Variable importance results for scenarios 5–6: the first two rows from the top to the bottom represent scenario 5 while the last two represent scenario 6; ∘ represents 10 noise variables, *Δ* 100 noise variables and + 500 noise variables; “LR” represents the adjusted Logrank splitting criterion and “R2” the Pseudo-R2 splitting criterion

**Fig. 9:**
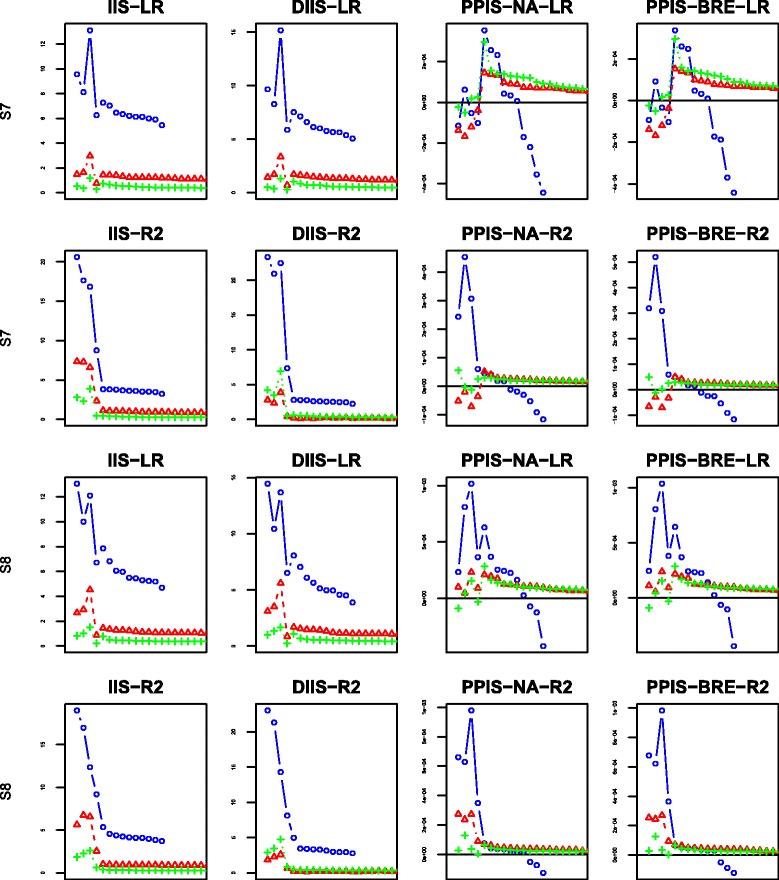
Variable importance results for scenarios 7–8: the first two rows from the top to the bottom represent scenario 7 while the last two represent scenario 8; ∘ represents 10 noise variables, *Δ* 100 noise variables and + 500 noise variables; “LR” represents the adjusted Logrank splitting criterion and “R2” the Pseudo-R2 splitting criterion

For each scenario and each proposed splitting criterion, we have computed four importance scores indexes: IIS, DIIS, PPIS-NA, PPIS-BRE. The behaviors of the four indexes are displayed in Figs. 6, 7, 8 and 9 using the mean over 50 replicates. Each figure displays the results obtained with the different number of additional noise variables: the blue color with the mark “ ∘” represents the case of 10 additional noise variables; the red color with the mark “ *Δ*” is set for 100 additional noise variables; the green color with marks “ + ” is set for the case of 500 additional noise variables. For the sake of readability of the figures, the first 4 dots for each color graph represent the scores associated with explanatory variables *G*_2_,*G*_3_,*G*_4_,*G*_5_, respectively, whereas the remaining dots are for noise variables ranking in decreasing order (for clarity, we only plot the first 20 ordered variables).

Figure 6 shows that in the simple Scenarios 1a and 2a with only 10 noise variables (blue color within Fig. 6), the pseudo-R2 splitting criterion attempts a clear discrimination between explanatory variables and noise variables, regardless of the considered importance scores. The same remark can not be made for the Logrank splitting criterion where the PPIS index discriminates only one variable while the IIS and DIIS indexes attempted to discriminate three explanatory variables from the noise ones.

In the more challenging scenarios 1b and 2b with 100 noise variables (red color within Fig. 6), the PPIS behaves poorly with the Logrank splitting criterion while the pseudo-R2 splitting criterion behaves well in discriminating the explanatory variables from the 100 noise variables. Nevertheless, the performances are quite similarly between the two splitting criterion with regard to IIS and DIIS.

In the most challenging scenarios 1c and 2c with 500 noise variables (green color within Fig. 6) we observe a little deterioration of performances, mainly for the PPIS index. The Logrank splitting criterion behaves poorly for all the indexes, while the IIS and DIIS for the pseudo-R2 splitting criterion still attempts a discrimination at low level compare to the previous ones.

The results of scenarios 3–4 are displayed in Fig. 7, where almost the half of the population is nonsusceptible. Combining this amount of plateau with censoring observations results in very few events observed in the scrutinized population. Compare to the previous scenarios, the results are quite similar for the pseudo-R2 splitting criterion with indexes IIS and DIIS. Nevertheless, the figure suggests a decrease in performances for indexes PPIS-NA and PPIS-BRE. Overall the Logrank splitting criterion performs very poorly regardless of the indexes.

The results of scenarios 5–6 are displayed in Fig. 8. These scenarios give more power for identify explanatory variables than the previous scenarios 1–4, since there is an increase in the population size by a factor of 2. As expected, the results are slightly better than all the results of scenarios 1–4. The pseudo-R2 splitting criterion allows a clear discrimination between the noise variables and the explanatory ones despite a very little degradation for PPIS indexes when the censoring rate increases and the number of noise variables is very high.

The results of scenarios 7–8 are displayed in Fig. 9. These results are quite similar to those of scenarios 5–6 for the pseudo-R2 criterion, mainly for the IIS and DIIS indexes despite the increase of the fraction of nonsusceptible individuals. Also, the PPIS performs poorly with a high number of noise variables.

**Fig. 10:**
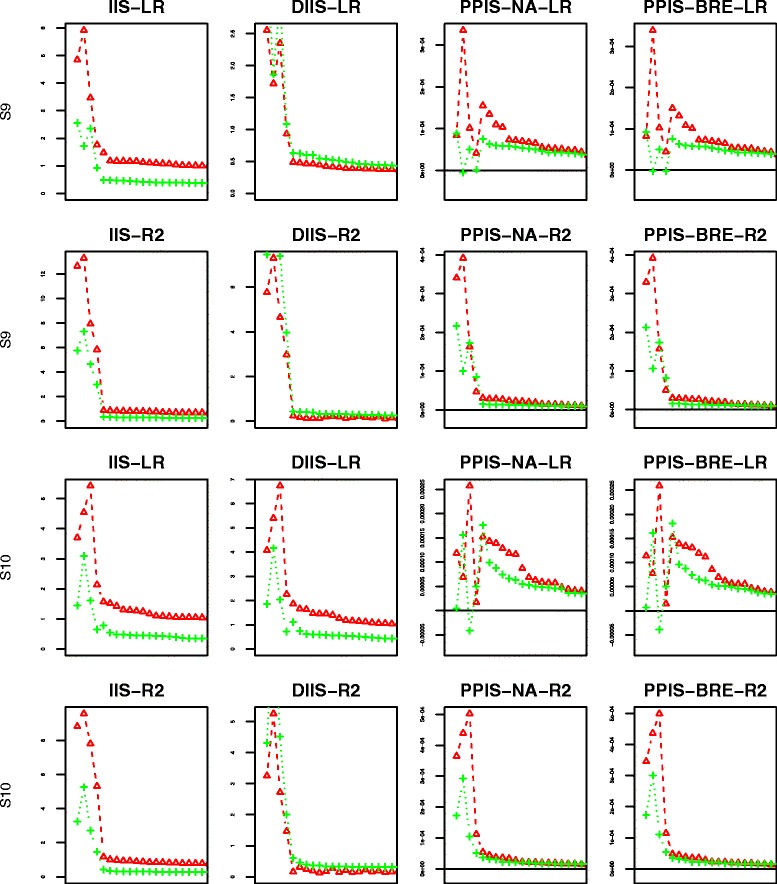
Variable importance results for the additional scenario with correlated variables: the first row from the top to the bottom represents a censoring rate of 10 *%* while the last row represents a censoring rate of 25 *%*; *Δ* represents 100 noise variables and + 500 noise variables; “LR” represents the adjusted Logrank splitting criterion and “R2” the Pseudo-R2 splitting criterion

The results of the additional scenario mimicking a data set that would reflect a situation such as the one presented in our example are displayed in Fig. 10. The pseudo-R2 splitting criterion attempts a clear discrimination between associated variables and noise variables for all the proposed criteria. The Logrank splitting criterion still has a poor performance for the PPIS indexes.

We investigated other scenarios with different values for the parameters related to the explanatory variables that lead to the same trends (results not shown). We also analyzed a scenario (results not shown) with a very small plateau value (5 %). As expected, our procedure outperforms the adjusted Logrank splitting method in terms of prediction accuracy but these gains are smaller than those obtained for higher “plateau” value. This is not surprising since the adjusted Logrank criteria can be seen as the limiting case of our criteria in which all the patients are susceptible. Thus, large power gains are anticipated in a situation where a non-negligible fraction of nonsusceptible patients is expected. However, if the plateau value is very small but identical for all individuals, then the classical unadjusted Logrank criteria should be more efficient.

### Analysis of breast cancer data

#### Description of the data

We used bio-clinical data extracted from two genomic datasets (GSE2034, GSE2990) publicly available on the GEO (Gene Expression Omnibus) website (http://www.ncbi.nlm.nih.gov/geo/). The GSE2034 dataset corresponds to the expression microarray study conducted by Wang et al. [28] and the GSE2990 dataset to the one conducted by Sotiriou et al. [29]. Both studies investigate the prognostic effect of gene expression changes on the outcome of patients with primary breast cancer. For gene expression analyses, Affymetrix Human Genome U133A Array were used in both studies and estrogen-receptor (ER) status (positive/negative) was available. The clinical outcome considered was the distant metastasis-free survival. Distant metastasis-free survival was defined as the interval from the date of inclusion to the first occurrence of metastasis or last follow-up.

For these two early breast cancer series, surgical resection can be considered as effective at eliminating the tumor burden for a non-negligible proportion of patients whereas, for the others, it leads to a lower tumor burden and thereby prolonged survival without distant relapse. Thus, a nonsusceptible fraction exists, and having a large number of patients followed up more than a decade after the primary treatment allows for an interpretable time sequence for tumor relapse.

For this work, we decided to investigate the impact of estrogen-related genes in predicting metastasis among patients with ER-positive tumors.

The gene expression datasets of the two series were analyzed after a joint quantile normalization. Here, we focused on estrogen-related genes that were defined as those demonstrating, on the whole dataset, a significant gene expression changes between ER-positive and ER-negative for a familywise error rate of 1 *%* (Bonferroni procedure). This selection led to the selection of 1,265 genes. We then selected patients with ER-positive tumors with a total of 294 patients (209 from GSE2034 and 85 from GSE2990) and investigated the effect of estrogen-related genes on the occurrence of distant metastasis.

In order to take into account the difference in the proportion of nonsusceptible patients between the two series, we included this variable as a confounding variable.

#### Results

**Fig. 11:**
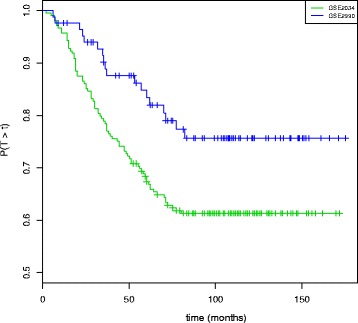
Kaplan-Meier Survival for the breast cancer data

Figure 11 displays the Kaplan-Meier estimate of the metastasis-free survival for the two series. The five year metastasis-free survival was 68.4 *%* ([*CI*_95 *%*_:62.3−75.0]) and 84.8 *%* ([*CI*_95 *%*_:77.2−93.1]) for the GSE2034 and GSE2990, respectively. It shows that the survival curve eventually reaches a plateau at seven years of 61.3 *%* ([*CI*_95 *%*_:55.0−68.3]) for GSE2034 and 75.7 *%* ([*CI*_95 *%*_:66.4−86.3]) for GSE2990.

**Fig. 12:**
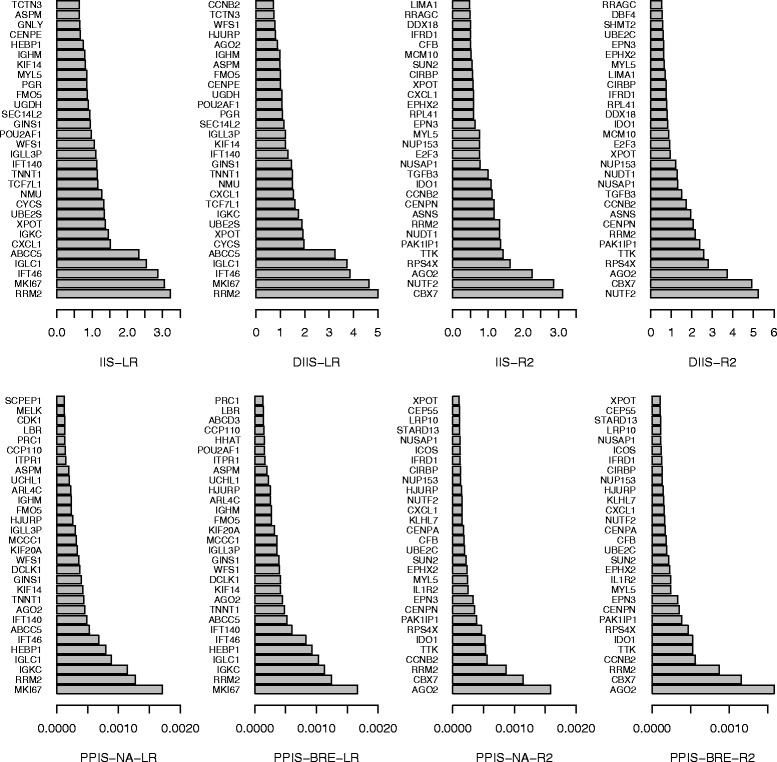
Importance variable score for the breast cancer data

We applied our proposed bagging survival procedure (with LR and R2 criterion) with 400 trees on the joint dataset presented just above. As can be seen from Fig. 12, the two splitting criteria lead to two different set of variables with very few overlap. As expected from the simulation results, for each splitting criteria, IIS, DIIS and PPIS give quite similar results.

**Fig. 13:**
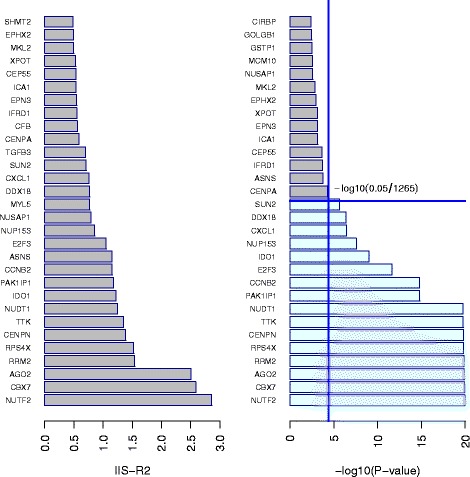
Basket selection based on IIS for the breast cancer data

The basket of important variables (based on the IIS importance score) obtained using the procedure selection presented previously leads to select 16 variables for both the pseudo-R2 and the adjusted Logrank criteria (see Fig. 13).

When looking to the first ten genes, no gene was selected in common between the adjusted Logrank and the pseudo-R2 criterion. The first five top-genes selected with the pseudo-R2 criterion are: CBX7, NUTF2, AGO2, RPS4X and TTK.

The CBX7 (Polycomb protein chromobox homolog 7) gene is involved in several biologic processes and recent works indicate a critical role in cancer progression. A relationship between the down-regulation of CBX7 expression and the tumor aggressiveness and poor prognosis has been reported in different cancer. Preliminary studies also indicate a potential role in the modulation of response to therapy [30].

The NUTF2/NTF2 (nuclear transport factor 2) gene encodes a small binding protein. The main function of NTF2 is to facilitate transport of certain proteins into the nucleus. It is also involved in regulating multiple processes, including cell cycle and apoptosis.

The AGO2 (Argonaute 2) gene is a central component of RNA-induced silencing complex which plays critical roles in cancer process through proliferation, metastasis and angiogenesis. AGO2 has been found over-expressed in various carcinomas and associated with tumor cell growth and poor prognosis [31].

The RPS4X (X-linked ribosomal protein S4) gene is involved in cellular translation and proliferation. Low RPS4X expression has been shown to be associated with poor prognosis in bladder, ovarian and colon cancer. Level of RPS4X is also a good indicator for resistance to platinum-based therapy and a prognostic marker for ovarian cancer. More recently, RPS4X has been identified as a partner of the overexpressed multifunctional protein YB-1 in several breast cancer cells. Depletion of RPS4X results in consistent resistance to cisplatin in such cell lines [32].

TTK (threonine tyrosine kinase, also known as Mps1) gene is essential for alignment of chromosomes to the metaphase plate and genomic integrity during cell. TTK gene has been identified as one of the top 25 genes overexpressed in tumors with chromosomal instability and aneuploidy [33]. TTK is overexpressed in a various solid cancers, and elevated levels of TTK correlate with high histological grade in tumors and poor patient outcome.

**Fig. 14:**
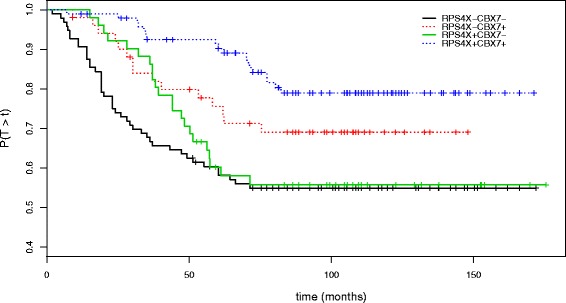
Survival for the breast cancer cross-stratified by CBX7 and RPS4X

In our analysis, we observed the marginal deleterious effects on distant relapse free survival of high expression of TTK, AGO2, NUTF2 and low expression of CBX7 and RPS4X. The Fig. 14 shows a clear negative prognostic effect of low levels of gene expression for CBX7 and RPS4X genes among patients with ER positive breast tumors. This finding is in accordance with published results than have exclusively focused either on CBX7 or RPS4X genes. The fact that these two markers are not selected when using the Logrank as splitting criteria is not surprising since we can observe a marginal non-proportional time-varying effect of RPS4X. This trend is probably linked to the time-dependent changes in the composition of the population since the fraction of susceptible patients is progressively exhausted as time goes on.

**Fig. 15:**
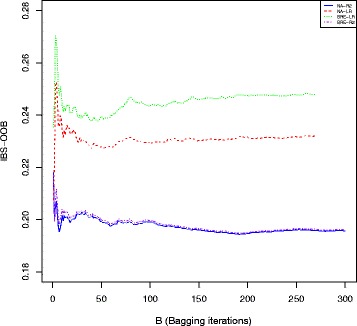
Out Of Bag Integrated Brier Score evolution with the number of Bagging iterations: NA-LR and BRE-LR represents the adjusted Logrank splitting method with the Nelson-Aalen and the Breslow estimates respectively; NA-R2 and BRE-R2 are obtained using the Pseudo-R2 splitting method

**Fig. 16:**
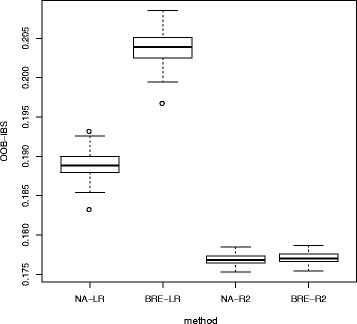
Box-plots of the Out Of Bag Integrated Brier Score for Breast Cancer data

In order to evaluate the variability of the results, we performed the same bagging procedure 50 times with 400 trees for each run. We then obtained 50 estimates of the Out Of Bag IBS for each method. Figure 15 shows the evolution of the OOB-IBS with the number of trees used in one random selected run of the bagging procedure for the four different procedures. It shows that 150 trees is clearly enough to stabilize the bagging predictor for all the criteria. As shown in this Figure, the procedure relying on the pseudo-R2 splitting criterion consistently outperforms the adjusted Logrank splitting method in terms of prediction accuracy. This result is further confirmed in Fig. 16, where the Box-plots of the 50 OOB-IBS are presented for all the procedures.

We also examined the Importance scores and 50 estimates of the importance scores for each procedures were computed. The mean of the 50 values is presented in Fig. 12 for the top 30 variables.

## Discussion

The discovery and predictive use of event-related markers have to face two main challenges that are the search over markers acting in complex networks of interactions and the potential presence of nonsusceptible patients in the studied population. In this work, we proposed a new bagging survival procedure devoted to this task. The strategy relies on an improper survival modelisation which considers a linear part for taking into account for known confounders associated with the nonsusceptible fraction and a tree structure for the event-related explanatory variables. The proposed tree-structured modeling differs from the tree-augmented Cox proportional hazards model proposed by Sun et al. [34] in that it is explicitly tailored for mixture population. Moreover, our procedure relies on the use of a splitting criteria which can be interpreted as a time-to-event discrimination index suited to mixed population.

The results of our simulation study show the good behavior of our bagging procedure based on the pseudo-R2 criterion as compared to the one relying on the classical Logrank statistic. For prediction, even though differences between the procedures are small, better predictions were obtained with the proposed procedure. If a difference between the fractions of nonsusceptible individuals is expected then the estimators that use the Breslow estimate should be preferred over those using the Nelson-Aalen estimate.

For variable selection, even in the presence of a high number of nuisance variables, our procedure is able to select the explanatory variables. The performance is obviously better when the number of events which can occur among susceptible patients is increasing. Based on our simulation study, we recommend the IIS or the DDIS criteria. These criteria rely on the discriminative performance of each splitting variable with or without the information related to the depth of the split. By contrast, the PPIS criterion which relies on prediction error is highly dependent on the censoring rate and the number of noise variables. Moreover, it is well-known that there is no consensus on which prediction error criterion should be used for survival data.

The search for markers that predict distant relapse in hormone receptor-positive treated patients is still an intensive area of study. In the analysis of the two series of early-stage breast cancer presented in this article, the proposed procedure is particularly appealing since the majority of the patients are amenable to cure and then will never recur from the disease. The fraction of nonsusceptible patients being clearly different between these two studies, we consider the study as a confounder variable. We obtain a selection of top-genes which is different from those obtained with the classical Logrank statistic. The five top genes selected with our procedure are related to cancer and most of them have only been recently reported to be associated with prognosis. In breast cancer, we know that various pathways related to the tumor process are activated and that there is no unique selection of prognostic factors. However, since our main aim is to select the more powerful set of predictors and obtain the highest prediction, our procedure should be preferred. This model-based selection which takes into account the high-order interactions and focuses on susceptible patients shed light on new markers that could serve as potential drug targets for new therapies.

In this work, we assumed that the hazard functions for the susceptible individuals between two child nodes are conditionally proportional given the node but the proportionality for any two nodes from different parents is not required. Postulating a proportional hazards structure within the whole tree could be an option which requires further development and evaluation. Here, we also considered the case with known confounding variables which is frequently encountered in biomedical research. For a different purpose, we could however consider extending the procedure to unknown confounding variables. Further works are however needed to cope with the potential degree of non-identifiability between failure time distribution of susceptible individuals and the proportion of nonsuceptibles individuals.

## Conclusion

In the presence of a mixed population with nonsusceptible patients, our results show that our bagging survival procedure with the proposed splitting criterion has good performance for prediction and variable selection. For measuring variable importance, we recommend the use of either the proposed Index Importance Score or the Depth and Index Importance Score.

The proposed tree-building process, which relies on a model-based splitting criteria, can be considered as a convenient hybrid solution that combines multiplicative intensity model and tree-structured modeling. We believe that the proposed survival bagging procedure is very appealing for many clinical genomic studies in which a fraction of nonsusceptible individuals is commonly encountered. This procedure has been implemented in a R package called *iBST* (improper Bagging Survival Tree) and will be available soon on the CRAN repository.

## Endnotes

Not applicable.

## Abbreviations

CART, classification and regression tree; CHF, cumulative hazard function; DIIS, depth and index importance score; ER, estrogen receptor; GEO, gene expression omnibus; IBS, integrated brier score; iBST, improper Bagging Survival Tree; IIS, index importance score; LR, log rank; OOB, out of bag; OOB-IBS, out of bag integrated brier score; PPIS, permutation prediction importance score; PPIS-NA, permutation prediction importance score with Nelson Aalen; PPIS-BRE, permutation prediction importance score with Breslow.

## References

[1] Breiman L, Olshen JH, Stone CJ (1984). Classification and Regression Trees.

[2] Gordon L, Olshen R (1985). Tree-structured survival analysis. Cancer Treat Rep.

[3] Bou-Hamad I, Larocque D, Ben-Ameur H (2011). A review of survival trees. Stat Surv.

[4] Davis RB, Anderson JR (1989). Exponential survival trees. Stat Med.

[5] LeBlanc M, Crowley J (1992). Relative risk trees for censored survival data. Biometrics.

[6] Hothorn T, Lausen B, Benner A, Radespiel-Tröger M (2004). Bagging survival trees. Stat Med.

[7] Ishwaran H, Kogalur UB, Blackstone EH, Lauer MS. Random survival forests. Ann Appl Stat. 2008;:841–60.

[8] Leblanc M, Crowley J (1993). Survival trees by goodness of split. J Am Stat Assoc.

[9] Shimokawa A, Kawasaki Y, Miyaoka E (2016). A comparative study on splitting criteria of a survival tree based on the cox proportional model. J Biopharm Stat.

[10] Maller RA, Zhou S (1995). Testing for the presence of immune or cured individuals in censored survival data. Biometrics.

[11] Tsodikov A, Ibrahim J, Yakovlev A (2003). Estimating cure rates from survival data. J Am Stat Assoc.

[12] Cooner F, Banerjee S, Carlin BP, Sinha D. Flexible cure rate modeling under latent activation schemes. J Am Stat Assoc. 2007; 102(478). 10.1198/016214507000000112 PMC2964090 21031152

[13] Rouam S, Broët P (2013). A discrimination index for selecting markers of tumor growth dynamic across multiple cancer studies with a cure fraction. Genomics.

[14] Fleming TR, Harrington DP (2011). Counting Processes and Survival Analysis vol. 169.

[15] Lin DY, Wei LJ (1989). The robust inference for the cox proportional hazards model. J Am Stat Assoc.

[16] Breslow N (1972). Discussion on ‘regression models and life-tables’(by dr cox). J Roy Statist Soc Ser B.

[17] Breslow N (1974). Covariance analysis of censored survival data. Biometrics.

[18] Nelson W (1972). Theory and applications of hazard plotting for censored failure data. Technometrics.

[19] Nelson W (1969). Hazard plotting for incomplete failure data. J Qual Technol.

[20] Korn EL, Simon R (1990). Measures of explained variation for survival data. Stat Med.

[21] Altman DG, Royston P (2000). What do we mean by validating a prognostic model?. Stat Med.

[22] Graf E, Schmoor C, Sauerbrei W, Schumacher M (1999). Assessment and comparison of prognostic classification schemes for survival data. Stat Med.

[23] Chen X, Liu CT, Zhang M, Zhang H (2007). A forest-based approach to identifying gene and gene–gene interactions. Proc Natl Acad Sci.

[24] Jiang H, Deng Y, Chen HS, Tao L, Sha Q, Chen J, Tsai CJ, Zhang S (2004). Joint analysis of two microarray gene-expression data sets to select lung adenocarcinoma marker genes. BMC Bioinforma.

[25] Diaz-Uriarte R, Alvarez de Andrés S. Gene selection and classification of microarray data using random forest. BMC Bioinforma. 2006; 7(3). 10.1186/1471-2105-7-3 PMC1363357 16398926

[26] Genuer R, Poggi JM, Tuleau-Malot C (2010). Variable selection using random forests. Pattern Recogn Lett.

[27] Altmann A, Toloşi L, Sander O, Lengauer T (2010). Permutation importance: a corrected feature importance measure. Bioinformatics.

[28] Wang Y, Klijn J, Zhang Y, Sieuwerts A, Look M, Yang F, Talantov D, Timmermans M, Meijer-van Gelder M, Yu J, Jatkoe T, Berns E, Atkins D, Foekens J (2005). Gene-expression profiles to predict distant metastasis of lymph-node-negative primary breast cancer. Lancet.

[29] Sotiriou C, Wirapati P, Loi S, Harris A, Fox S, Smeds J, Nordgren H, Farmer P, Praz V, Haibe-Kains B (2006). Gene expression profiling in breast cancer: understanding the molecular basis of histologic grade to improve prognosis. J Natl Cancer Inst.

[30] Pallante P, Forzati F, Federico A, Arra C, Fusco A (2015). Polycomb protein family member cbx7 plays a critical role in cancer progression. Am J Cancer Res.

[31] Ye Z, Jin H, Qian Q (2015). Argonaute 2: A novel rising star in cancer research. J Cancer.

[32] Garand C, Guay D, Sereduk C, Chow D, Tsofack SP, Langlois M, Perreault È, Yin HH, Lebel M (2011). An integrative approach to identify yb-1-interacting proteins required for cisplatin resistance in mcf7 and mda-mb-231 breast cancer cells. Cancer Sci.

[33] Carter SL, Eklund AC, Kohane IS, Harris LN, Szallasi Z (2006). A signature of chromosomal instability inferred from gene expression profiles predicts clinical outcome in multiple human cancers. Nat Genet.

[34] Su X, Tsai CL (2005). Tree-augmented cox proportional hazards models. Biostatistics.

